# Two GnRH-mitoxantrone Conjugates, Con-3 and Con-7, Target Endometrial Cancer Cells

**DOI:** 10.2174/0118761429343090250121052955

**Published:** 2024

**Authors:** Christos Markatos, Georgia Biniari, Vlasios Karageorgos, Oleg G. Chepurny, Maria Venihaki, George G. Holz, Theodore Tselios, George Liapakis

**Affiliations:** 1 Department of Pharmacology, School of Medicine, University of Crete, Heraklion, Greece; 2 Department of Chemistry, University of Patras, 26504 Rion, Pátrai, Greece; 3 Department of Medicine, State University of New York (SUNY), Upstate Medical University, Syracuse, New York 13210, USA; 4 Department of Clinical Chemistry, Faculty of Medicine, University of Crete, 71003 Heraklion, Greece; 5 Department of Medicine and Pharmacology, State University of New York (SUNY), Upstate Medical University, Syracuse, New York 13210, USA

**Keywords:** Endometrial cancer, Anticancer drugs, Gonadotropin-releasing hormone, Receptor, Mitoxantrone, Prodrugs

## Abstract

**Introduction:**

Endometrial cancer is one of the most common gynecological malignancies. Endometrial cancer cells express the gonadotropin-releasing hormone (GnRH) and its receptor (GnRH-R). Among the various therapeutic approaches for the treatment of endometrial cancer is the use of GnRH conjugates, such as the AN-152, created by linking the (D-Lys^6^) GnRH with the cytotoxic doxorubicin through an ester bond. An undesirable property of these conjugates is their vulnerability to plasma carboxylesterases, which cleave the ester bond to release doxorubicin before reaching the cancer cells.

**Methods:**

To overcome this problem, we recently developed the Con-3 and Con-7, which are GnRH analogs conjugated through a disulfide bond with the cytotoxic mitoxantrone. In this study, we determined the cytotoxic properties of the Con-3 and Con-7 on the Ishikawa endometrial cancer cells, assuming that their interaction with the GnRH-R of cells exposes the conjugated mitoxantrone to the cellular thioredoxin. The cellular thioredoxin reduces the disulfide bond of Con-3 & Con-7 to release mitoxantrone, which accumulates in the cancer cells and exerts its cytotoxic actions.

**Results:**

Indeed, treatment of Ishikawa cells with Con-3, Con-7, or the free unconjugated mitoxantrone increased their apoptosis and decreased their proliferation in a dose- and time-dependent manner, displaying half-maximal inhibitory concentrations (IC_50_) of 0.64 - 1.15 μM. In specific, the IC_50_ values on days 2, 3, and 4 were 1.45, 0.64, and 0.83 μM, respectively, for Con-3, 0.91, 0.82 μM, and 1.00 μM, respectively for Con-7 and 1.15, 0.98, 0.78 μM, respectively for mitoxantrone. In contrast, the free, mitoxantrone-unconjugated peptides did not affect the proliferation of Ishikawa cells.

**Conclusion:**

The Con-3 and Con-7 could put the basis for the development of a new class of anticancer drugs for endometrial cancer, which will act as “prodrugs” that deliver the cytotoxic mitoxantrone in a GnRH-R-specific manner.

## 1. INTRODUCTION

Endometrial cancer is one of the most common gynecological malignancies derived from the inner epithelial lining of the uterus [1, 2]. A therapeutic approach for the treatment of estrogen-dependent endometrial tumors is the use of long-acting GnRH I agonists, such as leuprolide, which act by downregulating the pituitary GnRH type 1 receptors (GnRH-I-R) and thus suppressing estrogen production, as well as, directly exerting antiproliferative effects on cancer cells [3 – 9]. In addition to the pituitary, GnRH-I-R is expressed in endometrial cancer cells, including the Ishikawa ones, regulating proliferation, cell cycle, and apoptosis [3, 5, 8, 10 – 15]. The direct action of GnRH agonists on GnRH receptor-expressing endometrial cancer cells is extremely important for the treatment of estrogen-independent endometrial ones, which do not respond to progesterone and estrogen analogs [2, 5]. In addition to GnRH-I-R, a second type of GnRH receptor, type 2 (GnRH-II-R), is expressed in many tumors [16, 17]. This receptor binds with relatively low affinity the GnRH I and its analogs and with high affinity the GnRH II, a second form of GnRH present in humans and many species [18]. However, although GnRH-II-R is a promising target to treat human cancers, including endometrial ones, it has not yet been used clinically, in contrast to GnRH-I analogs [5, 19, 20].

A milestone in the treatment of endometrial cancer and other cancer types expressing the GnRH-I-R, such as breast or ovarian ones, is the discovery of the AN-152, which is a GnRH I conjugate created by linking through an ester bond the [D-Lys^6^] GnRH with the cytotoxic doxorubicin [19, 21 – 23]. Interaction of the AN-152 with the GnRH-R in cancer cells results in the internalization of the receptor-conjugate complex, which releases cytotoxic doxorubicin in the intracellular environment. Injection of AN-152 into nude mice bearing endometrial cancer cells significantly reduced the tumor volumes [23]. AN-152 has entered clinical trials to treat GnRH-expressing endometrial cancer, as well as other tumors expressing GnRH receptors, such as breast and ovarian ones, showing promising results [19, 24]. In addition, other drug delivery systems, such as a GnRH-lytic peptide conjugate targeting endometrial and other cancers expressing GnRH receptors, have been developed [25]. In this conjugate, the targeting agent is the [D-Lys^6^] GnRH, and the loaded agent is the lytic peptide CLIP71.

However, AN152 is vulnerable to carboxylesterases of plasma, which release the doxorubicin before reaching the cancer cells by cleaving the ester bond between this cytotoxic agent and GnRH analog [26]. This disadvantage, along with the toxicity of doxorubicin, inspired us to develop the Con-3 and Con-7, which are modified leuprolide analogs conjugated through a disulfide bond with the antitumor agent, mitoxantrone [27]. The modified leuprolide analogs, which were used to create the Con-3 and Con-7, were nine amino-acid peptides named Con-P2 and Con-P1, respectively, and correspond to analogs 2 and 3, as described in detail in our previous study [27].

Mitoxantrone shows lower cardiotoxicity than doxorubicin and causes less vomiting, nausea, anorexia, diarrhea, stomatitis, and alopecia [28, 29]. Mitoxantrone is most likely accumulated within the cancer cells after the reduction of the disulfide bond linking this cytotoxic agent with the GnRH peptides. This chemical reaction is achieved by the thioredoxin system, which is overexpressed in cancer cells and reduces the disulfide bonds of proteins and peptides [30 – 32]. Although mitoxantrone has low activity against endometrial tumors, its conjugation to GnRH analogs likely increases its cytotoxic activity by concentrating it near the cell surface and exposing it to the cellular thioredoxin system for release [33]. Similarly, a formulation consisting of liposomes loaded with mitoxantrone has been proposed for treating endometrial cancer [34]. It is well known that tumor-selective drug delivery approaches based on the conjugation of cytotoxic agents with ligands targeting receptors overexpressed in cancer cells enhance the antitumor activity of anticancer drugs [35, 36].

## 2. MATERIALS AND METHODS

### 2.1. Synthesis of Con-3 and Con-7, GnRH-mitoxantrone Conjugates

The Con-3 and Con-7 were synthesized by our research team as previously described in detail [27]. Briefly, the nine amino-acid, modified leuprolide analogs, named Con-P2 (containing D-Cys^6^) and Con-P1 (containing D-Lys^6^), which correspond to analogs 2 and 3 of our previous study, respectively, were conjugated through a disulfide bond to mitoxantrone to create the Con-3 and Con-7 [27].

### 2.2. MTT Assay

The Ishikawa endometrial cancer cell line (Kindly provided by Prof. Kampa, University of Crete) was cultured into a 96-well plate at a density of 7000 cells/well in a humidified 5% CO_2_ incubator at 37°C in Dulbecco’s modified Eagle’s culture medium / F12 (DMEM/F12) (1:1), containing 3.15 g/L glucose and 10% bovine calf serum (BCS, HyClone). After an initial incubation of 24 hours, the culture medium was replaced by a fresh one containing 2% BCS, with or without increasing concentrations of Con-3, Con-7 (0.1nM – 10 μM), peptides, or mitoxantrone (0.1nM – 10 μM). The cells were incubated for 48, 72, and 96 hours in a humidified 5% CO_2_ incubator at 37°C. At the end of the incubation, 3-(4,5-dimethylthiazol-2-yl)-2,5- diphenyl tetrazolium bromide (MTT) reagent was added to each well at a final concentration of 0.5 mg/ml, and cells were incubated for 4 hours at 37°C in dark. A 4-hour incubation with the MTT reagent allowed the formation of formazan crystals by viable cells. At the end of the incubation, the MTT was removed, 100μl of isopropanol containing 4% HCl was added, and cells were incubated with shaking for 30 minutes in the dark. This procedure is required to dissolve the purple formazan crystals. At the end of the incubation, the absorbance of cells was measured at 594 nm using a Dynatech Micro Elisa reader (Chantilly, VA). The percentage of the proliferation rate (% proliferation rate) for each well was determined by multiplying by 100 the fraction absorbance of cells treated with a compound/absorbance of untreated cells. The percentage of viability of the untreated cells (control) was defined as 100%. The viability percentages were analyzed by nonlinear regression analysis, using Prism 8.0 (GraphPad Software, San Diego). The concentration of GnRH analogs or mitoxantrone that decrease cell proliferation by 50%, defined as half-maximal inhibitory concentration (IC_50_), was obtained by fitting the data using nonlinear regression analysis to a sigmoidal dose-response curve.

### 2.3. Cell Apoptosis Evaluation Using Annexin V and Propidium Iodide (PI)

Ishikawa cells were seeded into 12-well plates at a density of 50,000 cells per well 24 hours before the experiment in a humidified 5% CO_2_ incubator at 37°C. The culture medium was the DMEM/F12 (1:1), containing 3.15 g/L glucose and 10% BCS. The next day, the culture medium was replaced by a fresh one containing 2% BCS, with or without 1μM of Con-3, Con-7, peptides, or mitoxantrone. The cells were treated for 48, 72, and 96 hours at 37 °C and 5% CO_2_ and then harvested and collected into tubes together with their supernatant to avoid losing detached necrotic and apoptotic cells. The tubes were centrifuged at 300G for 5 minutes at room temperature, and the cells were washed twice with phosphate-buffered saline (PBS) (4.3 mM Na_2_HPO_4_7H_2_O, 1.4 mM KH_2_PO_4_, 137 mM NaCl, and 2.7 mM KCl, pH 7.3–7.4, at 37 °C). The cell pellets were resuspended in Annexin V binding buffer (supplied by Biotium, (CF488A Annexin V and PI Apoptosis Detection kit, Biotium, Cat. No. 30061) at a concentration of 80.000 - 100.000 cells/ml, and the mixtures were aliquoted into flow cytometry tubes at 100μl/per tube. Subsequently, the cells were incubated with the dyes Propidium Iodide (PI) at a concentration of 2 μl/tube and CF488A-conjugated Annexin V at a concentration of 5μl/tube, for 30 minutes in the dark, at 4°C. At the end of the incubation, 400μl of Annexin V binding buffer was added to each tube and immediately analyzed by flow cytometry on a BD FACS Calibur flow cytometer applying FITC channel parameters (filter 530 nm) for detection of the Annexin V-positive cells (Annexin V Ex/Em: 490/515 nm) and PI channel parameters (filter 616 nm) for detection of the PI-positive cells (PI Ex/Em: 530/622 nm). The data were analyzed using the FlowJo^™^ v10 software and presented as dot plot scattergrams showing the viable, necrotic, and apoptotic cells. Pro-apoptotic cells (cells that retain membrane integrity) were defined as Annexin V-positive and PI-negative (Q1 quadrant), apoptotic cells (cells with compromised membranes) were defined as double Annexin V/PI-positive cells (Q2 quadrant), necrotic cells were defined as PI-positive/Annexin V negative cells (Q3 quadrant) and intact cells as double Annexin V/ PI negative cells (Q4 quadrant). The percentage of apoptotic cells was determined by dividing the number of apoptotic cells by the total number of cells and then multiplying by 100.

### 2.4. Confocal Microscopy

Ishikawa cells, grown in DMEM/F12 (1:1) containing 1.15 g/liter glucose and 10% BCS at 37^o^C and 5% CO_2_, were seeded in 24-well plates at a density of 30.000 cells per well onto glass slides. The next day, the medium was replaced with a fresh one containing 2% BCS with or without 1μM of Con-3, Con-7, or mitoxantrone, and the cells were further incubated at 37°C and 5% CO_2_ for 2, 3, and 4 days. To block the thioredoxin reductase activity, cells were treated with its inhibitor, cisplatin (30μg/ml) 1 hour before the addition of Con-3, Con-7, or mitoxantrone. Next, the cells were washed with PBS and fixed using 4% paraformaldehyde (PFA). The cell nuclei were stained with the DNA-staining Hoechst 33342 dye (neoFroxx, Cat. No. 2289GR001) at a 1:10,000 dilution [37]. Coverslips were then mounted onto glass slides using 80% glycerol. Confocal fluorescent images were obtained using a Leica SP8 microscope and a 63X magnification lens. Sequential excitation at 350 nm (Hoechst dye) and 638 nm (mitoxantrone with dual-peak excitation maxima at 610 and 660 nm) was provided by the appropriate lasers [38]. Emission filters 461 nm and 650 nm - 700 nm were used for collecting blue (Hoechst dye) and red (mitoxantrone - emission maximum at 685 nm) signals in separate channels.

### 2.5. Statistical Analysis

To evaluate the significance of the antiproliferative effects of the GnRH-mitoxantrone conjugates Con-3 and Con-7 on Ishikawa cells, the results from the MTT experiments were analyzed using Prism 8.0 software. Nonlinear regression analysis was used to calculate the half-maximal inhibitory concentration (IC_50_) of the compounds by fitting dose-response data to a sigmoidal curve. Cell viability percentages were normalized to untreated control cells, defined as percentages, to assess the proliferation rates. Time- and dose-dependent effects were statistically analyzed using one-way ANOVA to compare treated and untreated cells, with a significance threshold of *p*<0.05. For flow-cytometry-derived apoptosis data, cell populations were categorized into viable, apoptotic, and necrotic subsets, and the percentage of apoptotic cells was calculated as a proportion of the total. Linear regression analysis and pairwise comparisons (Bonferroni test) using the SPSS (version 23, IBM) were performed on these results to evaluate the significance of the findings.

## 3. RESULTS

### 3.1. Antiproliferative Effects of Con-3 and Con-7 on Ishikawa Cells

To determine whether the Con-3 and Con-7 exert antiproliferative effects on Ishikawa cells, we incubated them for 2, 3, and 4 days with or without increasing concentrations of these GnRH-mitoxantrone conjugates. As observed in Fig. (1), the Con-3 decreased the proliferation rate of Ishikawa cells in a dose-dependent manner on days 2, 3, and 4 (Fig. 1A – C). The half-maximal inhibitory concentrations (IC_50_) of Con-3 were 1.45 ± 0.95 μM, 0.64 ± 0.03 μM; and 0.83 ± 0.33 μM, on days 2, 3 and 4, respectively (n=3 experiments). Similarly, the Con-7 inhibited the proliferation of Ishikawa cells in a dose-dependent manner, displaying IC_50_ of 0.91 ± 0.16 μM, 0.82 ± 0.20 μM, and 1.00 ± 0.19 μM on days 2, 3, and 4, respectively (n=3 experiments). The IC_50_ of Con-3 and Con-7 were similar to those of the free unconjugated mitoxantrone (1.15 ± 0.39 μM, 0.98 ± 0.13 μM, and 0.78 ± 0.15 μM, on days 2, 3, and 4 respectively, n=3 experiments) (Fig. 1A – C).

In marked contrast, as leuprolide, the peptidic ingredients of Con-7 and Con-3, Con-P1 and Con-P2, did not affect the proliferation of Ishikawa cells on days 2, 3, and 4 (Fig. 2).

In addition to the dose-dependent antiproliferative activity of the Con-3, Con-7, and mitoxantrone, these molecules decreased the growth of Ishikawa cells in a time-dependent manner, as observed in Fig. (3A). As expected, the leuprolide, Con-P1, and Con-P2 did not affect the proliferation of Ishikawa cells over time (Fig. 3B).

### 3.2. The Con-3, Con-7, and Mitoxantrone Induce Apoptosis in Ishikawa Cells

Subsequently, we determined the abilities of Con-3, Con-7, or mitoxantrone at concentrations of 1 μM to induce cell death. We accomplished this by selecting the percentages of live, apoptotic, and necrotic Ishikawa cells after treatment with the Con-3, Con-7, or mitoxantrone on days 2,3, and 4 (Fig. 4). On day 2, the majority of Ishikawa cells were viable (Q4 quadrant), whereas the percentage of apoptotic cells (early or late apoptotic) (Q1, Q2 quadrants), treated with Con-3, Con-7 or mitoxantrone, was only 15.75%, 13.11%, and 14.74%, respectively. On day 3, the percentage of apoptotic cells was 30.1%, 36.3%, and 32.7% after treatment with Con-3, Con-7, or mitoxantrone, respectively. Cell death further increased on day 4. In specific the percentage of apoptotic cells was 61.1%, 57.3%, and 57.8% after treatment with Con-3, Con-7, or mitoxantrone. Linear Regression analysis revealed that the percentages of apoptotic cells after their treatment with Con-3 were significantly different at different time points (*p*<0.001). Similar statistically significant results were obtained after treatments with Con-7 or mitoxantrone. On all days, the number of necrotic cells (Q3 quadrant) was very small.

Similar to their effects on cell proliferation, the unconjugated peptides, leuprolide, Con-P1, and Con-P2, did not reduce the viability of Ishikawa cells (Fig. 5).

### 3.3. Accumulation of Mitoxantrone within the Ishikawa Cells after their Treatment with Con-3, Con-7, or Mitoxantrone

The accumulation of mitoxantrone within the Ishikawa cells was determined with confocal microscopy, given that the mitoxantrone fluoresces [38]. In specific the excitation maxima of mitoxantrone are at 610 and 660 nm, whereas its emission maximum is at 685 nm. To demonstrate the nuclear localization of mitoxantrone, we stained the nuclei using the Hoechst dye, and we merged these images with those depicting the mitoxantrone fluorescence [37]. As observed in Fig. (6), mitoxantrone fluoresces in the cytoplasm and the nucleus of the Ishikawa cells after their treatment with 1 μM of Con-3, Con-7, or mitoxantrone for 6 hours. In contrast, the fluorescence signal was absent in untreated cells. Moreover, some nuclei in drug-treated cells showed a high staining density of Hoechst dye compared to untreated cells. This difference is expected and reflects chromatin condensation between untreated and treated cells. It is well known that apoptosis is related to a series of morphological and biochemical changes including chromatin condensation [39 – 41].

### 3.4. Cisplatin Inhibits the Intracellular Accumulation of Mitoxantrone of Con-3 and Con-7

The mitoxantrone of the Con-3 and Con-7 is most likely accumulated within the Ishikawa cells after the reduction of the disulfide bond linking the cytotoxic agent with the GnRH peptides. This chemical reaction is achieved by the thioredoxin system, which is overexpressed in cancer cells and reduces the disulfide bonds of proteins and peptides [30 – 32]. To verify this assumption, we treated the Ishikawa cells with the potent inhibitor of the thioredoxin system, cisplatin (30 mg/ml), for 1 hour before adding the Con-3 or Con-7 [42, 43]. As expected, the fluorescence red signal of mitoxantrone within the Ishikawa cells treated with Con-3 (image Con-3 + cisplatin) or Con-7 (image Con-7 + cisplatin) was reduced by cisplatin (Fig. 7). These results suggest that the intracellular accumulation of mitoxantrone was stopped by cisplatin.

Interestingly, cisplatin did not affect the intracellular accumulation of the free unconjugated mitoxantrone. Mitoxantrone is lipophilic, being able to enter the cell through its plasma membrane (Fig. 8).

## 4. DISCUSSION

In the present study, we determined the cytotoxic effects of the Con-3 and Con-7 on the endometrial adenocarcinoma cell line Ishikawa. The Con-3 and Con-7 were designed and synthesized by our research team by conjugating the leuprolide analogs Con-P1 and Con-P2 with the anticancer agent mitoxantrone *via* a disulfide bond [27]. These GnRH-mitoxantroneconjugates are bound to the GnRH-R with high affinity [27]. The GnRH-Rs of endometrial cancer cells are expected to attract the GnRH-mitoxantrone conjugates, which subsequently release the cytotoxic mitoxantrone into the cell. We verified this hypothesis by determining the antiproliferative effects of Con-3 and Con-7 on the Ishikawa cells. Con-3 and Con-7 decreased the proliferation rate of Ishikawa cells in a time- and dose-dependent manner. The larger effect of Con-3 and Con-7 at higher concentrations is likely attributed to the increased intracellular accumulation of the cytotoxic mitoxantrone released from the Con-3 and Con-7 by the cellular thioredoxin system. Based on the mass-action law, the higher the extracellular concentrations of Con-3 and Con-7, the faster the rate of their reaction with the GnRH-R of Ishikawa cells and thus their higher exposure to the cellular thioredoxin system will be.

The half-maximal inhibitory concentrations (IC_50_) of drugs were 1.45 μM, 0.64 μM and 0.83 μM for Con-3, 0.91 μM, 0.82 μM and 1 μM for Con-7 on the second, third, and fourth days of treatment. The free, unconjugated mitoxantrone had antiproliferative effects with similar IC_50_ with those of Con-3 and Con-7 (1.15 μM, 0.98 μM, and 0.78 μM second, third, and fourth days of treatment) and comparable to those in other cancer cells [44]. Similarly, the anticancer agent paclitaxel displayed an IC_50_ value in the micromolar range as Con-3, Con-7, and mitoxantrone (29.3±5.8 ug/ml or 35 μM) to decrease the proliferation of Ishikawa cells, [45]. Likewise, the IC_50_ values of cisplatin and carboplatin were 0.022-056 μg/ml (or 0.073-1.86 μM) and 0.096-1.2 μg/ml (or 0.26-3.23 μM), respectively in 8 different endometrial cancer cell lines [46]. In marked contrast, leuprolide and the unconjugated peptides Con-P1 and Con-P2 did not display antiproliferative effects on Ishikawa cells. Similar to endometrial cancer cells, the Con-3 and Con-7 exerted cytotoxic actions in the SKOV-3 ovarian cancer cells [47].

The antiproliferative effects of Con-3, Con-7, and free mitoxantrone were associated with their property to induce and promote apoptosis, as determined in flow cytometry experiments. Treatment of the Ishikawa cells with Con-3, Con-7, or mitoxantrone, elicited cell death mostly through apoptosis and, to a lesser extent, through necrosis. As in proliferative assays, the leuprolide and the free unconjugated GnRH analogs Con-P1 and Con-P2 did not elicit apoptotic death of the Ishikawa cells.

Although Con-3 and Con-7 exert cytotoxic actions *in vitro* in endometrial cancer cells grown as a monolayer and freely accessible to drugs, several considerations should be taken into account when assessing their *in vivo* cytotoxic effects on tumors, such as the absence of an immune response, differences between *in vitro* and *in vivo* receptor expression levels, elimination of reagents or their distribution into different compartments of the experimental animals’ body. Such parameters could contribute to potential differences between the *in vivo* and *in vitro* cytotoxic actions of the mitoxantrone-conjugated GnRH analogs.

These results suggest that the cytotoxic effects of the Con-3 and Con-7 were not due to their peptidic components, Con-P1 and Con-P2, but rather entirely to mitoxantrone, the cytotoxic mechanisms of which are known [44, 48, 49]. This assumption is further supported by confocal microscopy experiments, which demonstrated the intracellular accumulation of both free unconjugated and conjugated mitoxantrone of Con-3 and Con-7. It is most likely that the conjugated mitoxantrone of Con-3 and Con-7 within the Ishikawa cells arose from the cleavage of the compounds after the reduction of their disulfide bonds by the cellular thioredoxin system, which is overexpressed in cancer cells [30 – 32, 50]. This, in conjunction with the overexpression of the GnRH receptors in cancer cells, including the endometrial ones, likely contributes to increased accumulation of mitoxantrone in cancer cells compared to normal cells after its release from the Con-3 and Con-7 [25].

The thioredoxin system is located both intracellularly and extracellularly [30 – 32, 42, 50 – 53]. Inhibition of the thioredoxin system by cisplatin abolished the accumulation of the conjugated mitoxantrone of Con-3 and Con-7 within the Ishikawa cells [42, 43]. Similarly, the intracellular accumulation of anthraquinone, conjugated through a disulfide bond to the 85-99 epitope of the myelin basic protein, within the Jurkat cells was abolished by cisplatin [54]. These results suggest that the cleavage of the Con-3 and Con-7 was catalyzed by the extracellular thioredoxin system. The released mitoxantrone, as the free unconjugated one, are lipophilic molecules able to cross the plasma membrane of Ishikawa cells [55, 56]. In contrast, the uncleaved Con-3 and Con-7 are expected to be less lipophilic membrane-impermeable molecules.

It is less likely that the release of mitoxantrone from the Con-3 and Con-7 could be mostly attributed to the internalization of these GnRH-mitoxantrone conjugates in complex with the GnRH-R and reduction of the disulfide bond by the intracellular glutathione. In such a case, the intracellular fluorescence intensity of the conjugated mitoxantrone is expected to be smaller than that of its free unconjugated one. Based on receptor theory, only a small proportion of the added Con-3 or Con-7 is expected to interact with the GnRH-R [57]. However, the intracellular fluorescent intensities of both free and conjugated mitoxantrone were similar. In addition, the internalization of the human GnRH-R is a slow process due to the lack of a cytoplasmic carboxyl-terminal tail [58 – 62].

Although the agonist leuprolide and its analogs Con-P1 and Con-P2 bind to the GnRH-R with high affinities, they did not display cytotoxic properties. Similar to our results, the GnRH agonist, buserelin, and leuprolide were not cytotoxic in endometrial cancer cells and ovarian cancer, SKOV-3 cells, respectively [8, 63]. In contrast, previous studies have shown the GnRH agonists, leuprolide, goserelin, triptorelin, and decapeptyl GnRH, were cytotoxic for endometrial, ovarian, or prostate cancer cells [6, 9, 64 – 69]. These contradictory results could be due to the different signaling pathways stimulated by diverse conformational states of GnRH-R that are stabilized by different agonists, including those involving the stimulation of Gq and Gi proteins [59, 70 – 72]. This is supported by the fact that the substitution of Arg8 of GnRH I by Tyr, which corresponds to Tyr8 of GnRH II, decreased more than 30-fold the potency of the peptide to stimulate the Gq/IP3 generation, whereas increased 4-fold its potency to inhibit cell growth [59]. It is possible that Arg8 of GnRH-R stabilized the receptor in a conformational state associated with Gq/IP3 coupling. In contrast, the Tyr8 stabilized a receptor’s conformation with preferential Gi-mediated antiproliferative/apoptotic signaling [5, 59]. Molecular docking studies revealed that Arg8 of peptides interacts with Asp302 of the receptor, whereas Tyr8 likely contacts residues of the second extracellular loop of the receptor, thus stabilizing a different receptor conformation [59]. Different receptor densities in various cells could also have different impacts on the biological responses of even the same agonist [72]. For example, low doses of GnRH analogs were ineffective in SKOV-3 ovarian cancer cells, whereas they enhanced cell invasion in OVCAR-3 ovarian cancer cells, which express GnRH-R at higher levels [60].

Similar to Con-3 and Con-7, different conjugates of disulfide-linked anticancer agents with various ligands, such as folate, could act as prodrugs by releasing the cytotoxic agent in tumors after reduction of their disulfide bond. However, these conjugates exploit the intracellular glutathione, which is overexpressed in cancer cells, to reduce the disulfide bond after binding to their receptors in cancer cells and subsequent internalization of conjugate-receptor complexes [36, 73, 74]. Regardless of the reduction mechanism of the disulfide bond, its stability at physiological pH in plasma could confer all these prodrugs relatively less activity in healthy tissues, which is associated with reduced toxicity and increased target specificity. Specific linkers, including the disulfide ones, are also widely used to construct antibody-drug conjugates to treat cancer [75]. These linkers ensure the controlled delivery of the conjugates bearing the cytotoxic agent by prohibiting its release before reaching its target.

Future modifications might increase the cytotoxic activity of Con-3 and Con-7. For example, Con-3 and Con-7 could be linked with specific substances to construct nanocarriers, such as liposomes or dendrimers, that contain active ingredients, including siRNAs, antisense oligonucleotides, or combinations of different chemotherapeutic agents to suppress pump and non-pump resistance [76 – 79]. Such conjugates would be very useful in endometrial cancers resistant to chemotherapy due to the overexpression of drug efflux pump proteins and/or mutations of various proteins, such as β-tubulin [80, 81].The efficacy and safety of Con-3 and Con-7, as well as future modified counterparts of these GnRH-mitoxantroneconjugates, will be evaluated in *in vivo* experiments using a variety of experimental animals with endometrial cancers [82, 83].

## CONCLUSION

The interaction of the GnRH-mitoxantrone conjugates, Con-3 and Con-7, with the plasma membrane GnRH-R locally increased the concentration of mitoxantrone and exposed it to the extracellular thioredoxin system. Reduction of the disulfide bond of Con-3 or Con-7 by the thioredoxin system cleaves them to release the lipophilic mitoxantrone, which passively crosses the plasma membrane of cancer cells, thus exerting its cytotoxic effects. The GnRH-mitoxantrone conjugates, Con-3 and Con-7, act as “prodrugs” that deliver the cytotoxic mitoxantrone in a GnRH-R-specific manner. Con-3 and Con-7 could be used as lead compounds for the development of novel formulations, such as nanocarriers containing active ingredients, including sRNAs, antisense oligonucleotides, or combinations of different chemotherapeutic agents to suppress pump and non-pump resistance. Such formulations would be very useful clinically in endometrial cancers resistant to chemotherapy.

## Figures and Tables

**Fig. (1).**
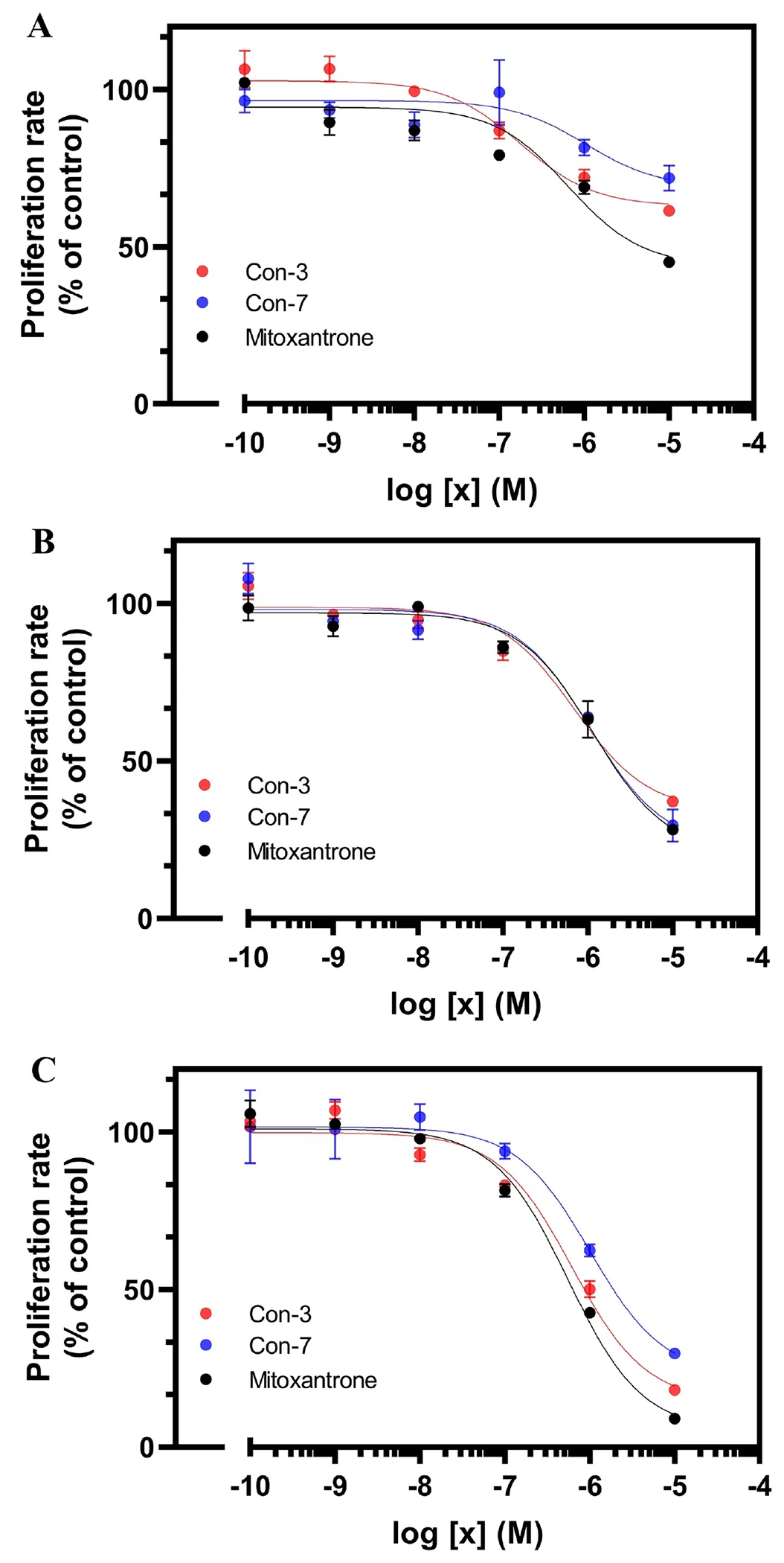
Antiproliferative actions of the Con-3, Con-7, and mitoxantrone. The proliferation rate of the Ishikawa cells (cultured into a 96-well plate at a density of 7000 cells/well) was decreased after their treatment with increasing concentrations (0.1nM – 10 μM) of Con-3, Con-7, or mitoxantrone for 2 days (**A**) 3 days (**B**), and 4 days (**C**) at 37 °C. At the end of the incubation, the cells were incubated with 0.5 mg/mL MTT for 4 h at R.T., and the formed purple formazan crystals were determined at the absorbance of 594 nm. The means ± S.E (triplicate determination) are shown from a representative experiment performed three times with similar results.

**Fig. (2).**
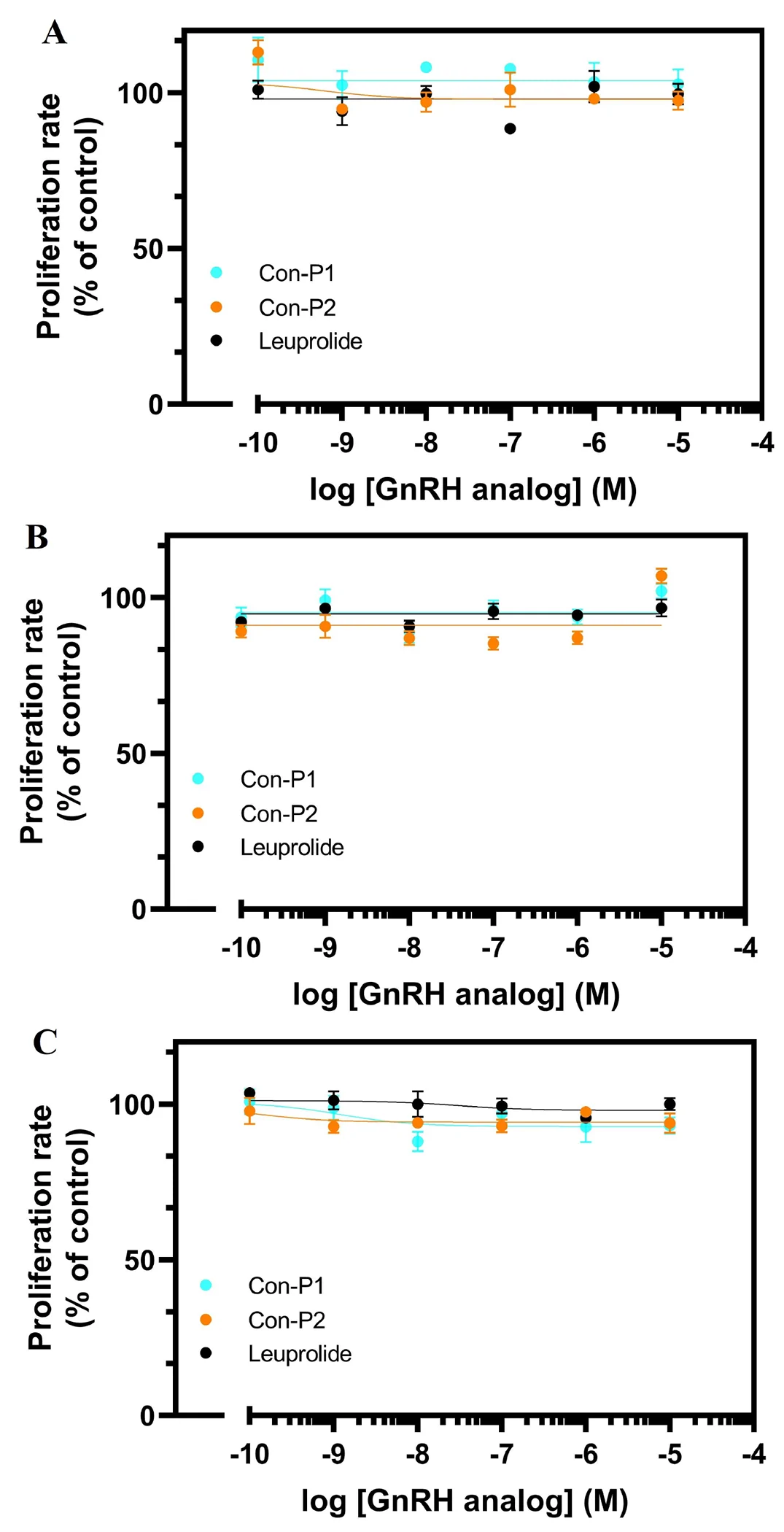
Effects of the GnRH peptides on the proliferation of Ishikawa cells. The Ishikawa cells (cultured into a 96-well plate at a density of 7000 cells/well) were treated with increasing concentrations (0.1nM – 10 μM) of Con-P1, Con-P2, or leuprolide for 2 days (**A**) 3 days (**B**), and 4 days (**C**), at 37 °C. The proliferation rate of cells was determined. At the end of the incubation, the cells were incubated with 0.5 mg/mL MTT for 4 h at R.T., and the formed purple formazan crystals were determined at the absorbance of 594 nm. The means ± S.E (triplicate determination) are shown from a representative experiment performed three times with similar results.

**Fig. (3).**
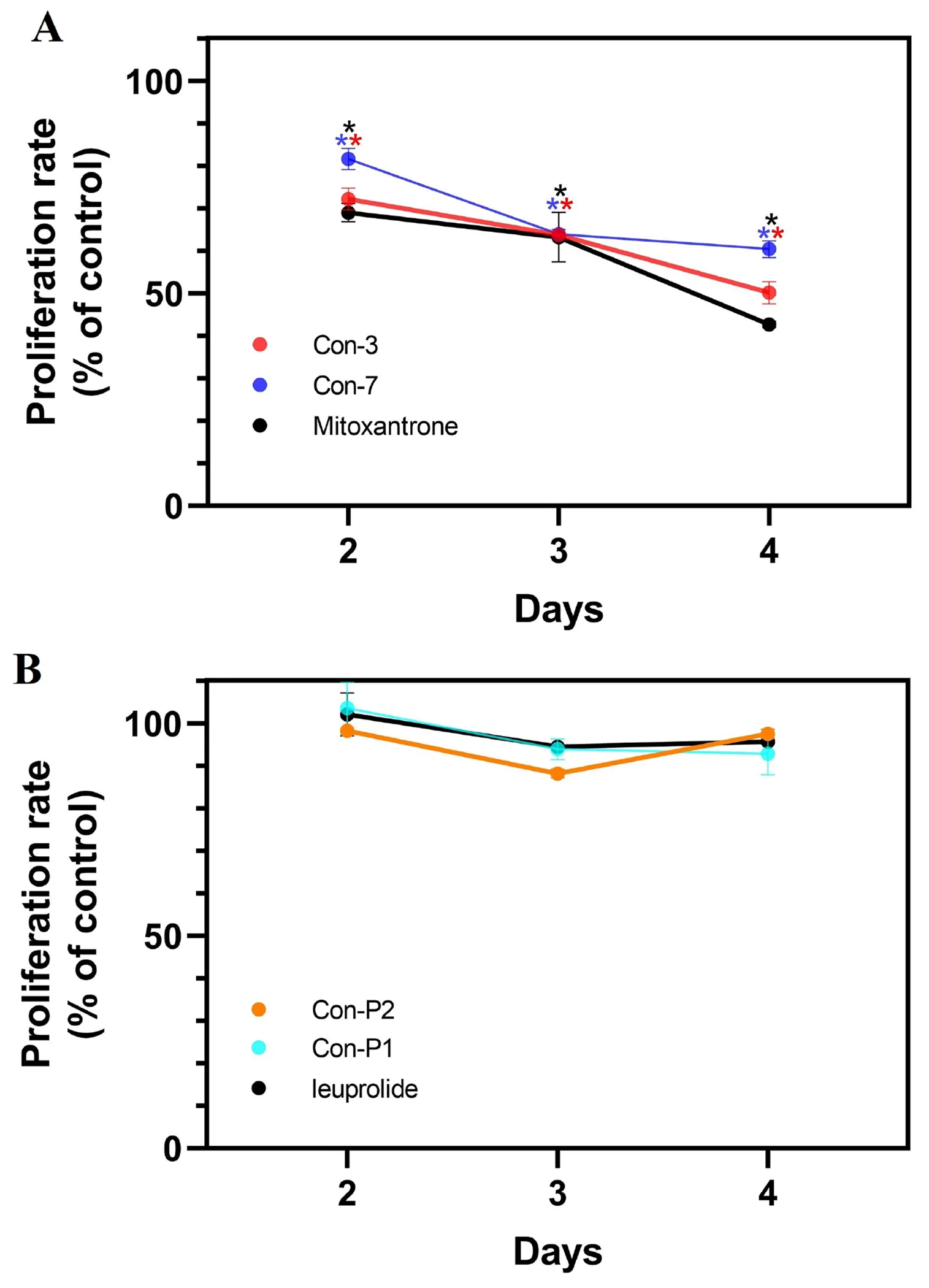
Time-dependent antiproliferative actions of the GnRH analogs and mitoxantrone. The proliferation rates of cells treated with 1 μM of GnRH analogs and mitoxantrone were used to plot graphs (**A** and **B**), which depict the decrease of cell growth over the incubation time. Statistical analysis was performed using one-way ANOVA. The asterisk symbols indicate that the proliferation rates of cells treated with Con-3, Con-7, or mitoxantrone are significantly different from those of untreated cells (controls) (*p* < 0.05). Data analysis and graph creation was performed using Prism 8.0 (GraphPad Software, San Diego). Statistical tests were performed using ver 23, SPSS, IBM software.

**Fig. (4).**
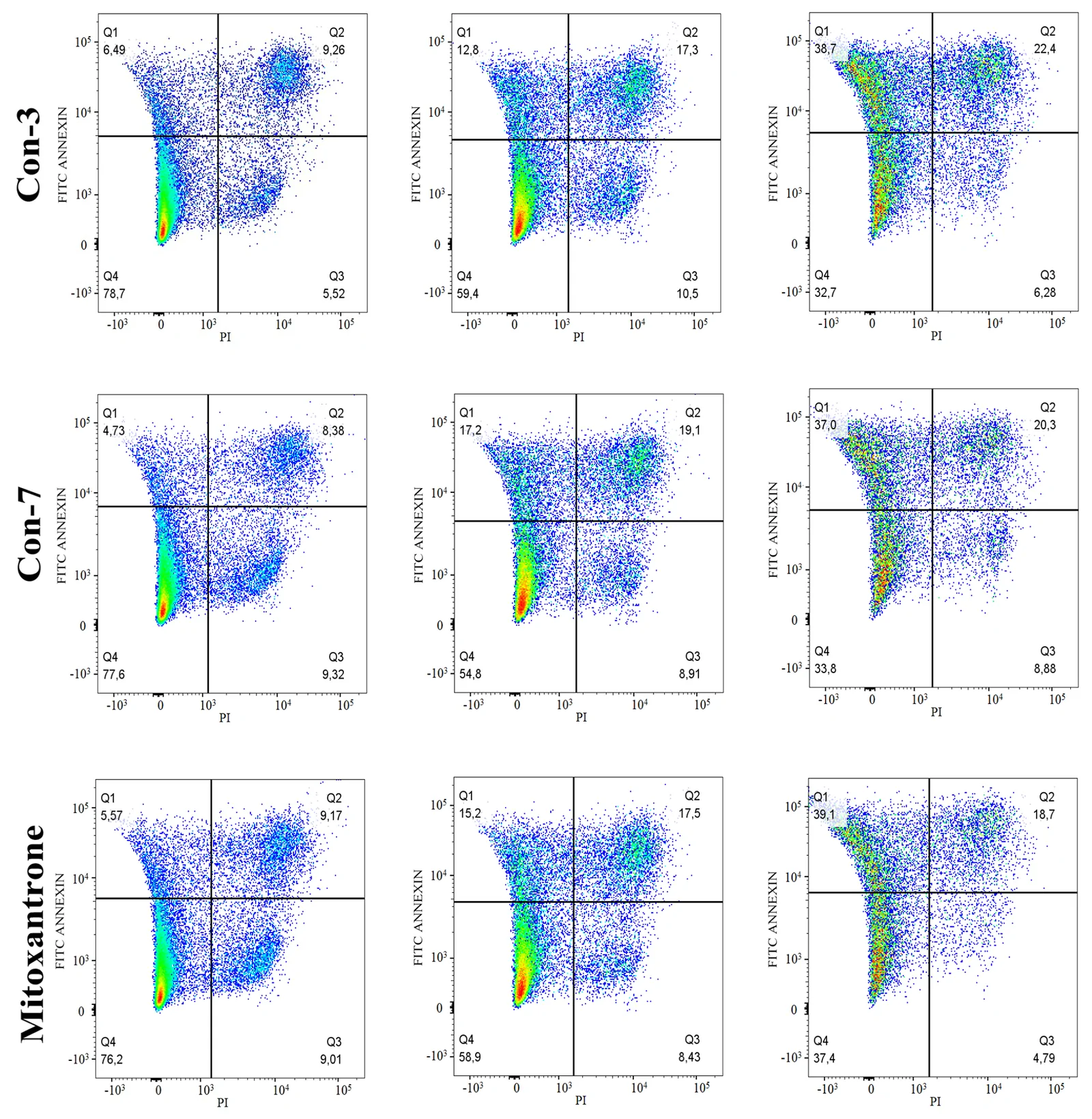
Determination of apoptosis, necrosis, or viability of Ishikawa cells (cultured into a 12-well plate at a density of 50,000 cells/well) after their treatment with 1μM Con-3, Con-7, or mitoxantrone for 2 (left), 3 (middle), and 4 (right) days at 37 °C. At the end of the incubation, the cells were incubated in the binding buffer of Biotium (CF488A kit) and analyzed on a flow cytometer (excitation 488 nm) in the FITC channel (filter 530 nm) for detection of the Annexin V-positive cells and in the PI, channel (filter 616 nm) for detection of the PI-positive cells. The figures are from a representative experiment performed three times with similar results.

**Fig. (5).**
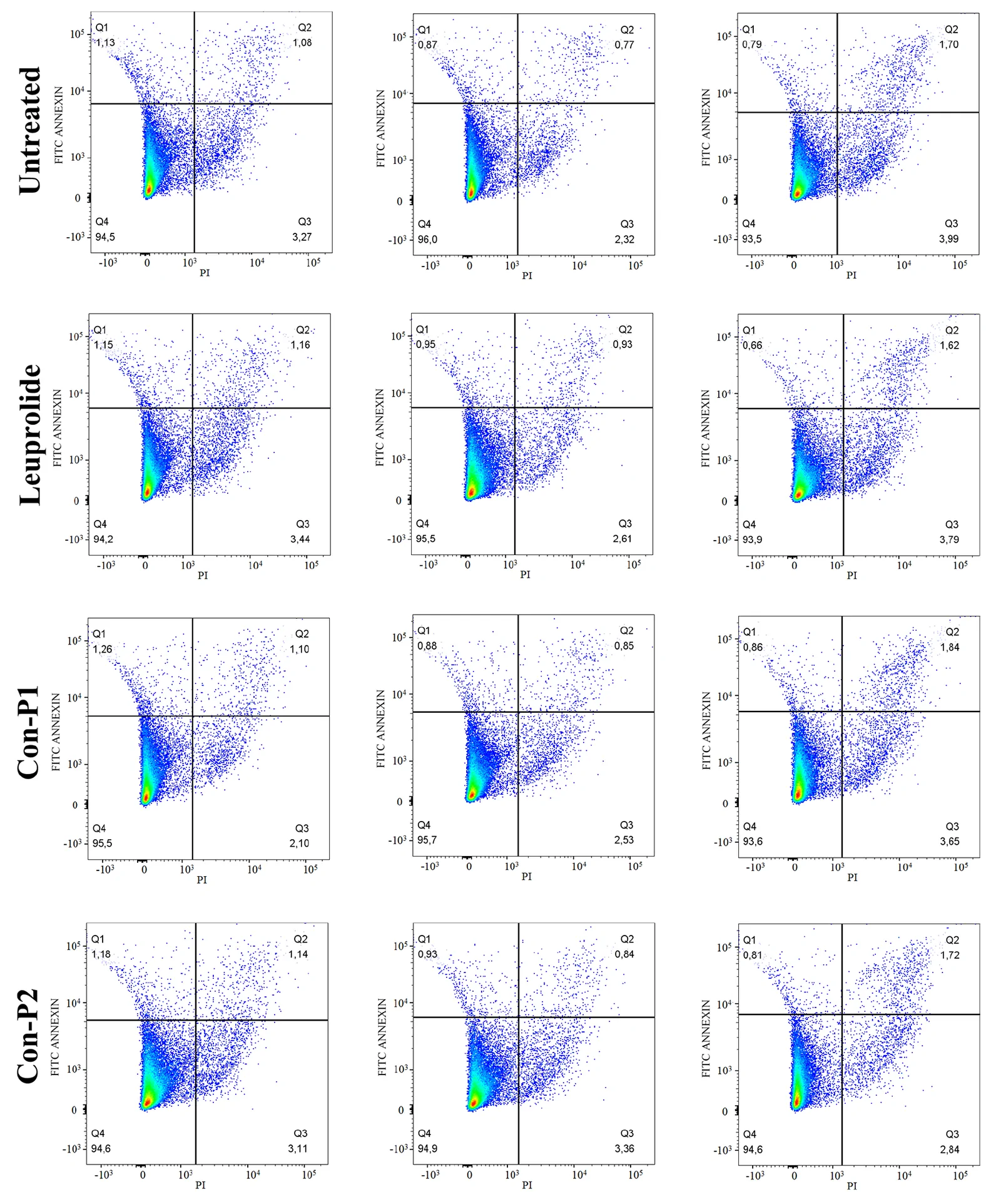
Determination of apoptosis, necrosis, or viability of Ishikawa cells (cultured into a 12-well plate at a density of 50,000 cells/well) after their treatment with 1 μM leuprolide, Con-P1, or Con-P2 for 2 (left), 3 (middle), and 4 (right) days at 37 °C. At the end of the incubation, the cells were incubated in the binding buffer of Biotium (CF488A kit) and analyzed on a flow cytometer (excitation 488 nm) in the FITC channel (filter 530 nm) for detection of the Annexin V-positive cells and in the PI, channel (filter 616 nm) for detection of the PI-positive cells. The figures are from a representative experiment performed three times with similar results.

**Fig. (6).**
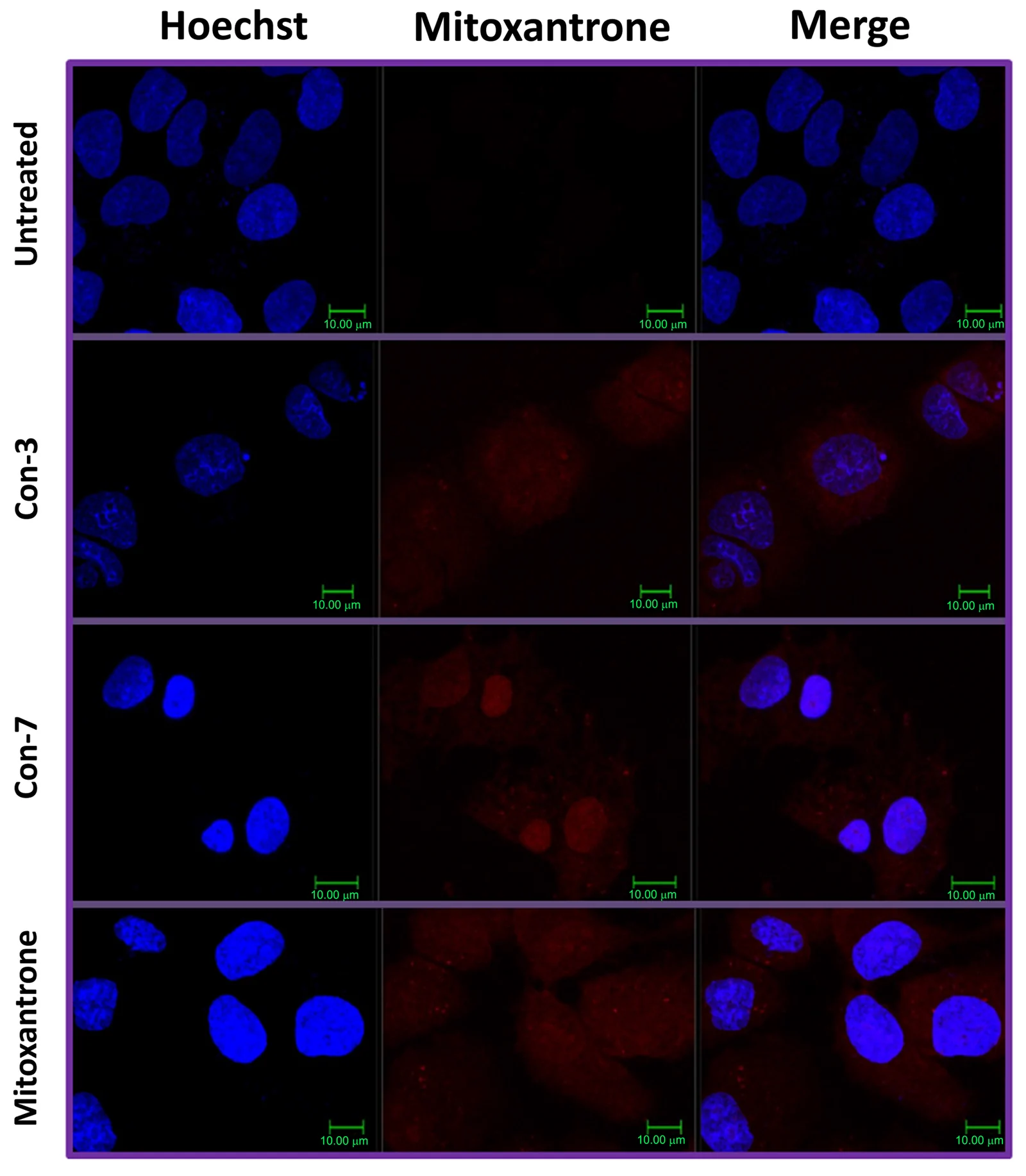
Confocal microscopy images of Ishikawa cells treated with or without 1μM Con-3, Con-7, or mitoxantrone for 6h at 37 °C. At the end of the incubation, the cell nuclei were stained with the DNA-staining Hoechst 33342 dye, and confocal fluorescent images were obtained after excitation at 350 nm (for the Hoechst dye) followed by excitation at 638 nm (for the mitoxantrone). The emission filters of 461 nm and 650 nm–700 nm were used for obtaining blue (Hoechst dye) and red (mitoxantrone) signals in separate channels. The left images show the nuclei staining with Hoechst dye (blue), whereas the middle ones depict the mitoxantrone fluorescence (red). The right images were created by merging the left and middle ones and depict the localization of mitoxantrone (red) in both the cytoplasm and the nuclei of the Ishikawa cells. The figures are shown from a representative experiment performed three times with similar results.

**Fig. (7).**
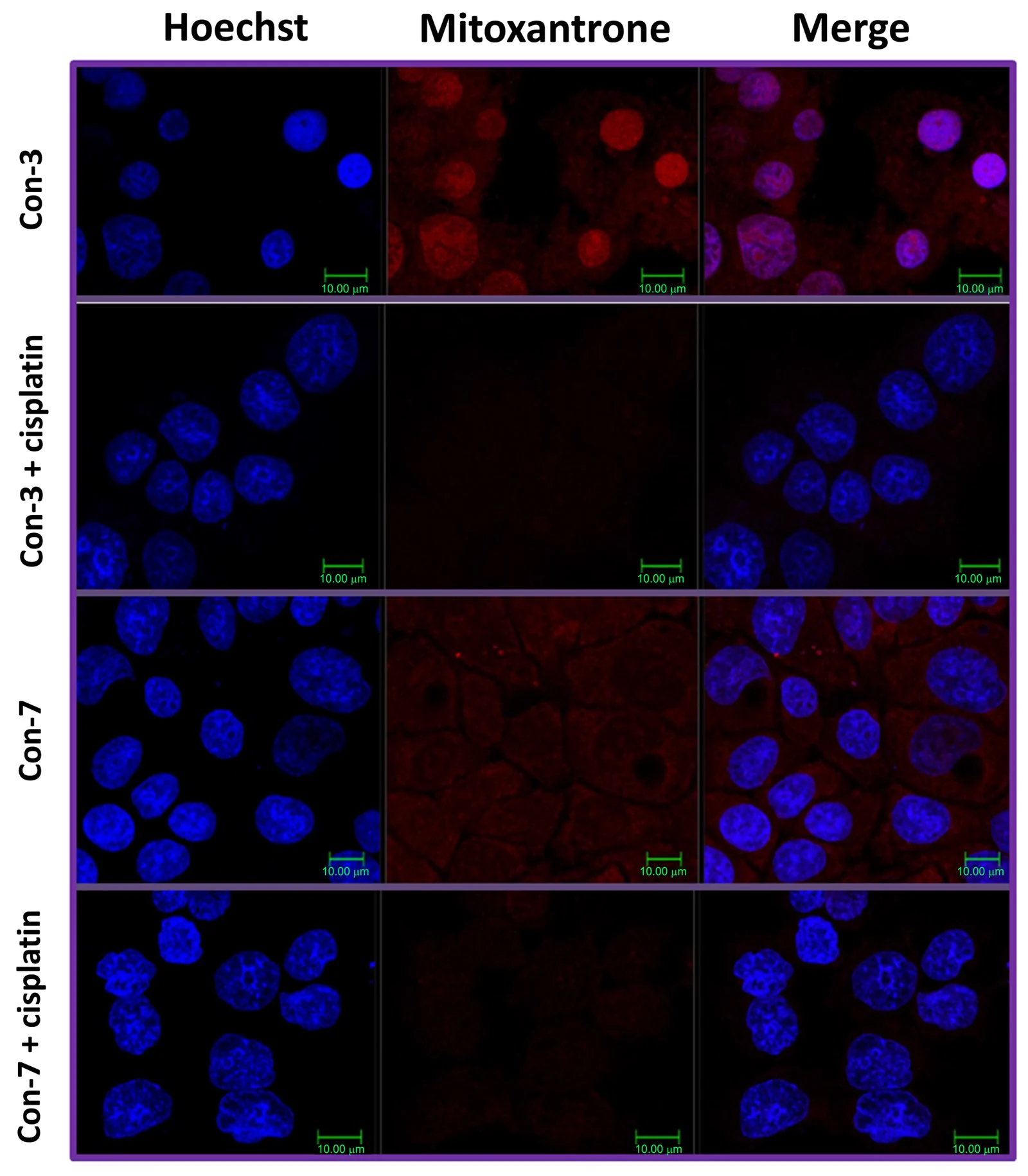
Confocal microscopy images of Ishikawa cells treated with the Con-3 or Con-7 (1μM for 6h) with or without 1-hour preincubation with cisplatin at 37 °C. At the end of the incubation, the cell nuclei were stained with the DNA-staining Hoechst 33342 dye, and confocal fluorescent images were obtained after excitation at 350 nm (for the Hoechst dye) followed by excitation at 638 nm (for the mitoxantrone). The emission filters of 461 nm and 650 nm–700 nm were used for obtaining blue (Hoechst dye) and red (mitoxantrone) signals in separate channels. The left images show the nuclei staining with Hoechst dye (blue), whereas the middle ones depict the mitoxantrone fluorescence (red). The right images were created by merging the left and middle ones and depict the localization of mitoxantrone (red) in both the cytoplasm and the nuclei. The figures are shown from a representative experiment performed three times with similar results.

**Fig. (8).**
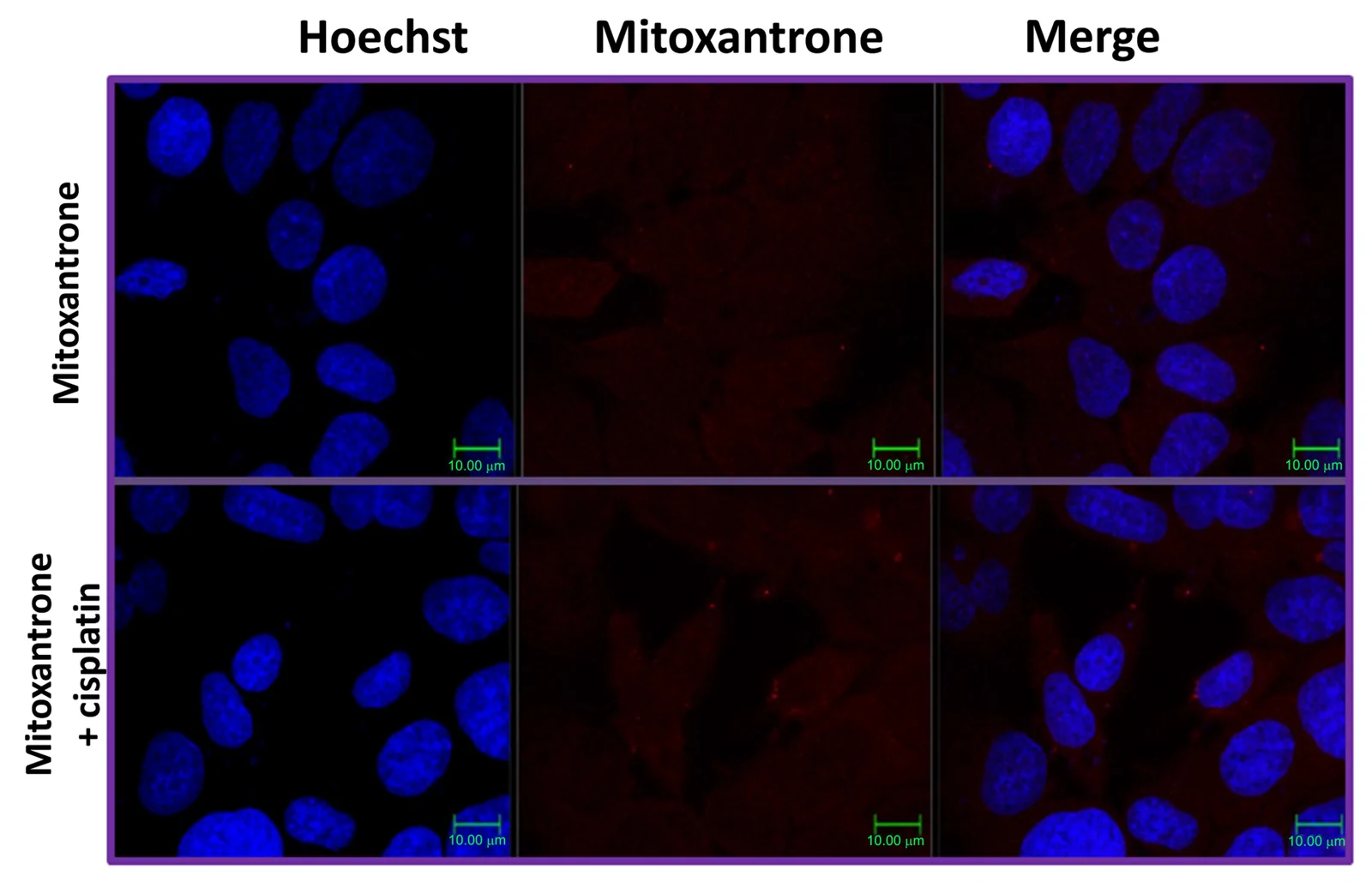
Confocal microscopy images of Ishikawa cells treated with the mitoxantrone (1μM for 6h) with or without 1-hour preincubation with cisplatin at 37 °C. At the end of the incubation, the cell nuclei were stained with the DNA-staining Hoechst 33342 dye, and confocal fluorescent images were obtained after excitation at 350 nm (for the Hoechst dye) followed by excitation at 638 nm (for the mitoxantrone). The emission filters of 461 nm and 650 nm–700 nm were used for obtaining blue (Hoechst dye) and red (mitoxantrone) signals in separate channels. The left images show the nuclei staining with Hoechst dye (blue), whereas the middle ones depict the mitoxantrone fluorescence (red). The right images were created by merging the left and middle ones and depict the localization of mitoxantrone (red) in both the cytoplasm and the nuclei. The figures are shown from a representative experiment performed three times with similar results.

## Data Availability

All the data and supporting information are provided within the article.

## LIST OF ABBREVIATIONS

GnRH: Gonadotropin-Releasing Hormone
GnRH-R: Gonadotropin-Releasing Hormone Receptor
GnRH-I-R: Gonadotropin-Releasing Hormone Type 1 Receptor
GnRH-II-R: Gonadotropin-Releasing Hormone Type 2 Receptor
IC50: Half-Maximal Inhibitory Concentration
DMEM/F12: Dulbecco’s Modified Eagle’s Medium/Nutrient Mixture F-12
BCS: Bovine Calf Serum
MTT: 3-(4,5-Dimethylthiazol-2-yl)-2,5-Diphenyltetrazolium Bromide
PBS: Phosphate-Buffered Saline
PI: Propidium Iodide
FITC: Fluorescein Isothiocyanate
PFA: Paraformaldehyde
ANOVA: Analysis of Variance
siRNAs: Silencing RNA
IC50: Half-Maximal Inhibitory Concentration
R.T.: Room Temperature
